# Learning Processes and Acquisition of Knowledge and Skills in Training and Supervision of Psychotherapy and Counselling: A Study Protocol for a Scoping Review

**DOI:** 10.3389/fpsyg.2021.718314

**Published:** 2021-12-16

**Authors:** Hanne Weie Oddli, Erkki Heinonen, Stephan Hau, Jan Nielsen, Rachelle Esterhazy, Cecilie Hillestad Hoff, Hanne Strømme

**Affiliations:** ^1^Department of Psychology, Faculty of Social Sciences, University of Oslo, Oslo, Norway; ^2^Finnish Institute for Health and Welfare, Helsinki, Finland; ^3^Department of Psychology, Faculty of Social Sciences, Stockholm University, Stockholm, Sweden; ^4^Department of Psychology, Faculty of Social Sciences, University of Copenhagen, Copenhagen, Denmark; ^5^Department of Education, Faculty of Educational Sciences, University of Oslo, Oslo, Norway

**Keywords:** competence, counseling, expertise, learning processes, psychotherapy, supervision, scoping review, training

## Abstract

**Background:** Increased awareness of the individual therapist’s vital contribution to treatment processes and outcome, and the potential role of training and supervision in this respect, warrants a close look at the empirical and theoretical literature on teaching and learning of therapists and counselors.

**Methods:** A scoping review of the literature will be conducted based on an overarching research question: when authors have reported on learning processes and acquisition of knowledge and skills in psychotherapy/counseling and supervision/training literature over the past 30 years (since 1990), what evidence, concepts, theories, and models have they reported? A comprehensive search strategy is carried out to identify publications indexed in Scopus, PsycINFO, and Cochrane Central Register of Controlled Trials. Publications will be sorted according to four categories: (1) conceptual/theoretical; (2) empirical (quantitative, qualitative, mixed methods); (3) review, meta-synthesis or -analysis; (4) training program/model description. Procedures for the upcoming scoping review of conceptual/theoretical, empirical, and training program/model description publications will be outlined.

**Conclusion:** Besides clarifying existing perspectives, practices, and evidence, and documenting the shifting trends of the field during the past three decades, this scoping review identifies knowledge gaps that point to vital future directions for research and theory development. Moreover, the comprehensive scoping lays the foundation for subsequent, more focused systematic reviews that address identified key research topics more specifically.

## Introduction

Increased awareness of the vital contribution of the individual therapist to treatment processes and outcome (e.g., Wampold and Imel, 2015; Castonguay and Hill, 2017) warrants a close look at the processes involved in clinicians’ knowledge and skills acquisition. How do clinicians develop clinical competence, and what learning processes are involved? Within the psychotherapy literature this question has been illuminated from the perspectives of *clinical training and supervision* (Hill and Knox, 2013; Hill et al., 2015; Watkins et al., 2015; Callahan and Watkins, 2018; Bernard and Goodyear, 2019), *therapist/professional development* (Stoltenberg et al., 1998; Rønnestad and Skovholt, 2003, 2013; Orlinsky and Rønnestad, 2005), and *expertise* (Tracey et al., 2014; Hill et al., 2017), each of which serves to complement the picture of what clinical competence involves and how it develops through postgraduate training and professional career.

Professional competence may be defined as “the habitual and judicious use of communication, knowledge, technical skills, clinical reasoning, emotions, values, and reflection in daily practice for the benefit of the individual and community served” (Epstein and Hundert, 2002, p. 226). Handling complexity is key, and a central characteristic, reflected in the literature and various definitions of competence, is the capacity to integrate various sources of knowledge – scientific and clinical – as well as knowledge modalities, such as analytic/declarative and procedural, explicit and implicit, conscious and intuitive modes of processing (e.g., Stoltenberg et al., 2000; Fraser and Greenhalgh, 2001; Rodolfa et al., 2005; Sharpless and Barber, 2009; Fairburn and Cooper, 2011). Due to the complex, contextual, and ever shifting nature of the situations in which clinical competence is called for, Fraser and Greenhalgh (2001) suggested that, besides ensuring competence, educators need to enable capability, that is, the “[e]xtent to which individuals can adapt to change, generate new knowledge, and continue to improve their performance” (Fraser and Greenhalgh, 2001, p. 799), which among other things involves relational and non-linear learning, based on practice and feedback throughout the training. These are central components of training in profession-oriented psychology/counseling programs, of which an overarching goal is to enable the development of clinical competence.

### Learning/Training Processes and Supervision

One of the main educational activities through which students and post-graduates are to develop clinical competence and expertise is clinical practice under the supervision of professional therapists. Although there is no consensus on a particular definition of supervision in the field, a comprehensive and widely used definition is that of Bernhard and Goodyear, stating that:

Supervision is an intervention provided by a more senior member of a profession to a more junior colleague or colleagues who typically (but not always) are members of the same profession. This relationship is evaluative and hierarchical, extends over time, and has the simultaneous purposes of enhancing the professional functioning of the more junior person(s); monitoring the quality of professional services offered to the clients that she, he, or they see; and serving as a gatekeeper for the particular profession the supervisee seeks to enter (Bernard and Goodyear, 2014, p. 9).

The core functions of supervision may be summed up as “(i) acquisition and improvement of therapeutic skills and knowledge; (ii) quality control and accountability to the client and to the public; (iii) transmission of the culture of psychotherapy, including ethical behavior; and (iv) professional development and growth” (Grant and Schofield, 2007, p. 3). Moreover, the relationship and the working alliance between supervisor and supervisee have attracted increased attention over the past decades (e.g., Watkins, 2014a; Watkins et al., 2015).

Supervision is demonstrably crucial for psychotherapists’ subjective experience of development (e.g., Orlinsky and Rønnestad, 2005; Wheeler and Richards, 2007; Hill and Knox, 2013; Rønnestad and Skovholt, 2013; Bernard and Goodyear, 2019), and constitutes the “signature pedagogy” of psychotherapy training through which students learn to think and act like professional therapists (Shulman, 2005; Watkins, 2014b; Baltrinic and Wachter, 2020). Despite ample evidence of the importance of supervision for psychotherapy training, the mechanisms of effective supervision – including students’ and candidates’ actual learning processes – are still largely unknown (Bernard and Goodyear, 2019). Thus, a question that still calls for elaboration, is how knowledge and skills acquisition come about, and what learning processes are involved.

### Aim

Scoping reviews are a form of knowledge synthesis particularly aimed at identifying available evidence, research methods, key concepts, definitions, and theories, including potential knowledge gaps in the literature, either for this purpose itself or as a precursor to a systematic review (Arksey and O’Malley, 2005; Levac et al., 2010; Munn et al., 2018; Tricco et al., 2018). In search for the prevailing knowledge on clinical training and supervision, as well as preparing for relevant recommendations for future research, this scoping review will be guided by an overarching research question: when authors have reported on learning processes and acquisition of knowledge and skills in psychotherapy/counseling and supervision/training literature over the past 30 years (since 1990), what evidence, processes, concepts, and theories have they reported? More specifically, we aim to identify: (1) Studied processes, concepts, and theories; (2) Applied research designs and methods; (3) Empirical evidence for given learning processes and knowledge/skills acquisition; (4) Existing meta-analyses, meta-syntheses, and literature reviews; (5) Descriptions of training models and programs.

## Method

### Literature Search

We are using a broad search strategy to cover the extensive literature on therapist training and supervision across the diverse terminology and various education practices in and between nations. The following electronic databases will be searched to identify relevant studies: Scopus (covers nearly 100% of MEDLINE titles), PsycINFO, and Cochrane Central Register of Controlled Trials. While all authors were involved in the decision on relevant terms, the particular search strategy was developed by the first and last author in consultation with a an experienced librarian by the [Name of library withheld for review] and revised according to the Peer Review of Electronic Search Strategies (PRESS) guideline (McGowan et al., 2016). See Supplementary Appendix A for description of terms and combinations chosen for the search in the included databases.

#### Review Inclusion Criteria

Based on titles and abstracts, the research group will identify publications that concern the topic of the scoping review, and exclude others. The software program for screening studies is Excel. Inclusion of publications will be based on the following decisions:

##### Conceptual and Empirical Studies

Our review will cover both empirical findings and the prevailing views among researchers of the mechanisms involved in learning and skills acquisition. Thus, conceptual as well as empirical studies will be included, as both are valuable sources of knowledge on learning processes and competence development.

##### Publications Aimed at Training and Supervision (Leaving Out Formal Lectures)

Clinical training and supervision that involve skills acquisition and the integration of knowledge with skills and values may be contrasted to formal graduate education and acquisition of knowledge (e.g., Falender et al., 2004). This division is blurred once we take into account how tightly intervowen these forms of knowing are in actual clinical practice and in clinical education programs. Procedural and theoretical/conceptual knowledge are intertwined and interdependent, and theoretical lectures likely influence the effects of skills training and vice versa, each forming vital parts of the integrated knowledge necessary to develop professional clinical competence (Cf. the scientist-practitioner model, e.g., Shapiro, 1967, 2002; Baker and Benjamin, 2000; Stoltenberg et al., 2000). To understand these development processes, this scoping review will include all publications aimed at training and supervision; however, studies that are focused on aspects of education not directed at applied professional knowledge, such as theoretical, scientific courses and lectures in an academic psychology program will be excluded.

##### Literature on Both Graduate and Postgraduate Training and Supervision

To capture a rich picture of training and learning during professionals’ careers we will include studies on training and supervision at all stages and phases. Moreover, education, training, and licensing standards for therapists vary across and within nations, with some countries emphasizing academic education and others postgraduate training programs. For maximal representativeness, we will include studies on both.

##### Literature on Both Teaching and Learning

The aspects of teaching and learning are often intertwined in the literature. Therefore, both will be reviewed to address questions of knowledge and skills acquisition.

##### Both Explicit and Implicit Accounts of Learning Processes and Acquisition of Knowledge and Skills

Empirical studies not explicitly investigating processes of learning or competence should be included, as they may nevertheless enable valuable inferences of presumed processes of learning and knowledge acquisition. This may, for example, be the case in surveys or clinical trials looking at the general features or effects of particular training programs.

##### Only Studies on Education and Supervision of Students Who Are Supposed to Conduct Psychotherapy or Counseling

Studies within adjacent disciplines may be included insofar as the treatment that students or professionals are trained for comprise psychotherapy or counseling. Studies of clinical training aimed at for example musicians, physicians, and occupational therapists who will not use the training for psychotherapy but rather within their own professional discipline will not be included, irrespective of how clinically oriented the training may be. Moreover, studies on medical training of psychiatrists will be excluded, even if medication may form part of the total psychosocial treatment.

##### Only Publications in English Will be Included

There will be no restrictions as to research settings.

Besides the above explicit guidelines for inclusion, we will be open to various forms of formal and informal training and supervision to make sure we include the different variants that exist in the broad field of psychotherapy and counseling.

Further, studies will be sorted and categorized into four broad categories: (1) *Conceptual/theoretical*, including journal publications, books and book chapters; (2) *Empirical*, including denotation of applied research design and methodology (i.e., quantitative, qualitative, and mixed methods design); (3) *Review, meta-synthesis* or *meta-analysis*; (4) *Training program/model description*. If a study fits in more than one category it will be classified and counted as such. We will exclude conference abstracts, conference papers, opinions, personal views, unpublished doctoral theses, editorials, commentaries, and publications that lack abstracts. See the eligibility screening form in Supplementary Appendix B.

#### Selection of Studies

As an initial step of including and excluding publications the research group of five reviewers will read a subset of the abstracts to ensure agreement upon the selection criteria. Inter-rater reliability will be evaluated. Finally, the total number of publications will be equally distributed among the group of researchers. Consequently, each of the five reviewers, based on reading the study titles and abstracts, will select publications in their range, according to the above criteria for inclusion, and sort them into the four categories. In case of uncertainty of classification and potential inconsistencies, these will be brought into discussion and resolved by discussions among the group members. Further quality checks will also be made along the way into each reviewer’s ratings to ensure similar rating process and any inconsistencies or disagreements resolved similarly through discussion. In line with the remit of scoping reviews, quality assessment of all included publications will not be conducted (Arksey and O’Malley, 2005; Levac et al., 2010; Munn et al., 2018).

#### Charting of Data

After selection and sorting of publications, data will be charted, which means that information from the included studies will be extracted and sorted into a data-charting form (e.g., Levac et al., 2010; Peters et al., 2015). The research team will engage in an iterative, collaborative process to ensure extractions that are relevant to the research questions.

#### Collating, Summarizing and Reporting of Results

A thematic analysis (Braun and Clarke, 2006) and narrative synthesis of the findings will be conducted in relation to the research questions, ensuring a comprehensive picture of the prevailing and shifting trends of concepts, theories, models, and evidence of how clinicians develop clinical competence, and what learning processes are involved. For the empirical articles we will identify themes, whereas for the remaining publications we will present an overview. Moreover, in order to provide an overview of prevailing and shifting trends of publications, we will quantify the prevalence of different kinds of articles (conceptual, empirical, reviews/meta-syntheses and meta-analyses, and training program descriptions) in different decades.

A flow chart depicting the steps of the review decision process will be presented, including the numerical results from the identification and selection of studies, removal of duplicates and the final presentation. The reporting will be guided by the PRISMA extension for scoping reviews checklist (the PRISMA-ScR; Tricco et al., 2018).

## Discussion and Conclusion

Theoretical and empirical literature on training and supervision of therapists and counselors span a heterogeneous and evergrowing field of norms and practices, conceptualizations and definitions, as well as research methods and designs. The literature covers various approaches to both psychotherapy and its training and supervision, reflected in different curriculae and training programs within and across nations. Research efforts have been noted to be diverse, characterized by varying methodological quality (Hill and Knox, 2013). The planned scoping review is warranted to identify key conceptualizations and definitions, empirical approaches, and available evidence, and will help planning further systematic reviews to pursue more specific questions in this heterogeneous field. Besides clarifying existing perspectives, practices, and evidence, a broad scoping review will identify knowledge gaps pointing to vital future directions for research and theoretical development.

## Limitations

The current scoping review will only cover studies published in English, and will therefore not be fully representative of the literature. This is a field where there might be a substantial body of work published at a national level, and we may miss important studies reflecting the prevailing knowledge among researchers, practitioners, trainers, and supervisors in various nations. Further, the planned review also excludes gray literature, such as editorials, commentaries, policy papers, and unpublished theses. While excluding this literature may result in overlooking some of the discussions within the field, it is not expected to bias the main aim of the review, i.e., identifying the trends of the field over a long period of time and its central knowledge gaps.

Scoping reviews are typically aimed at identifying and mapping available knowledge rather than conducting in-depth analyses of the existing literature. Although this is within the aims of a scoping review (Arksey and O’Malley, 2005; Munn et al., 2018; Tricco et al., 2018) and therefore not a limitation in this genre, it is worth noting that the conclusions given cannot go into any depths. A scoping review may, however, function as a valuable first step and guide for further studies conducted by other forms of knowledge synthesis, such as systematic reviews.

## Ethics, Dissemination, and Impact

As the scoping review methodology implies identification and analysis of published literature, no ethical approval is required.

Results will have broad impact, spanning from the scientific community to the educational field and mental health care. The upcoming review is embedded in a multisite, multidisciplinary research program studying psychotherapy and psychotherapy training [name of study withheld for review]. This research consortium consists of researchers at universities in three Nordic countries, holding positions within the disciplines of psychology and higher education sciences. The findings will feed back into these institutions and training programs, and disseminated to scholars internationally through the researchers’ participation in the scientific community of training and psychotherapy. We will identify the under-researched and researched eras on training and supervision of therapists, and will mirror this in the existing literature on the characteristics of effective therapists (Heinonen and Nissen-Lie, 2020). This will help us inform therapist trainers as well as researchers on future avenues for developing effective therapists, and where more research is needed. Moreover, the scoping review will represent a point of departure for identifying more narrow areas and questions to guide systematic reviews of empirical research within this broad field. Along with publications in relevant scientific journals, network activities such as conferences, workshops and seminars will ensure dissemination of findings to various groups of professionals, consisting of scientists, lecturers, psychotherapy supervisors, students, and clinicians. As attainment of professional competence is relevant broadly beyond psychology and education sciences, the findings will be applicable and useful for profession-oriented education and programs in a variety of disciplines. The dissemination of results includes policy papers of relevant findings to inform stakeholders and policy makers about areas in need of more funding.

## Author Contributions

HO and HS responsible for the consultation with the Medical Library, University of Oslo, regarding the planning of the literature search. HO wrote the first draft of the manuscript. All authors contributed to manuscript revision, read, and approved the submitted version and conception and design of the study.

## Conflict of Interest

The authors declare that the research was conducted in the absence of any commercial or financial relationships that could be construed as a potential conflict of interest.

## Publisher’s Note

All claims expressed in this article are solely those of the authors and do not necessarily represent those of their affiliated organizations, or those of the publisher, the editors and the reviewers. Any product that may be evaluated in this article, or claim that may be made by its manufacturer, is not guaranteed or endorsed by the publisher.
