# Supplementary material for: Learning Processes and Acquisition of Knowledge and Skills in Training and Supervision of Psychotherapy and Counselling: A Study Protocol for a Scoping Review
Source: Front Psychol. 2021 Dec 16;12:718314. doi: 10.3389/fpsyg.2021.718314 (PMC8716550; doi:10.3389/fpsyg.2021.718314)
Supplement: Supplementary file 1 [file Presentation_1.pdf]

Appendix A

We are conducting a search within the following electronic databases to identify relevant studies:

- Scopus (covers nearly 100 % of MEDLINE titles)
- PsycINFO
- Cochrane Central Register of Controlled Trials

Terms and Combinations for the Search in the Included Databases

Scopus (Elsevier). The search sentences below are combined as follows:
(1 AND 2) **OR** (3 AND 4 AND 5)
1
TITLE (psychother* OR counsel*)
2
TITLE (supervis* OR trainee* OR training)
3
TITLE-ABS-KEY (psychother* OR therap*)
4
TITLE (psychotherapist* OR psychologist* OR psychiatrist* OR therapist* OR professional* OR clinician* OR counsellor* OR counselor* OR personnel* OR employee* OR practioner* OR supervisor*)
5
TITLE (supervis* OR counseling OR counselling OR tutor* OR mentor* OR guiding OR guidance OR trainee* OR placement* OR training OR praxis* OR teach* OR skill* OR knowledge OR competen* OR performanc* OR learn* OR educat* OR qualificat* OR "professional development" OR "therapist development" OR "therapist's development" OR career* OR "personal practice" OR "clinical practice" OR ((development OR developing) AND practice*) OR "practice setting" OR "practice settings" OR experienc*))
Limits: articles in English language, published from 1990 - .

All search sentences above are combined with AND/OR into this search strategy. Copy + paste into Scopus (advanced > enter query string):

TITLE ((psychother* OR counsel*) AND (supervis* OR trainee* OR training)) OR (TITLE-ABS-KEY (psychother* OR therap*) AND TITLE (psychotherapist* OR psychologist* OR psychiatrist* OR therapist* OR professional* OR clinician* OR counsellor* OR counselor* OR personnel* OR employee* OR practioner* OR supervisor*) AND TITLE (supervis* OR counseling OR counselling OR tutor* OR mentor* OR guiding OR guidance OR trainee* OR placement* OR training OR praxis* OR teach* OR skill* OR knowledge OR competen* OR performanc* OR learn* OR educat* OR qualificat* OR "professional development" OR "therapist development" OR "therapist's development" OR career* OR "personal practice" OR "clinical practice" OR ((development OR developing) AND practice*) OR "practice setting" OR "practice settings" OR experienc*)) AND (LIMIT-TO (LANGUAGE , "English")) AND (LIMIT-TO (PUBYEAR , 2019) OR LIMIT-TO (PUBYEAR , 2018) OR LIMIT-TO (PUBYEAR , 2017) OR LIMIT-TO (PUBYEAR , 2016) OR LIMIT-TO (PUBYEAR , 2015) OR LIMIT-TO (PUBYEAR , 2014) OR LIMIT-TO (PUBYEAR , 2013) OR LIMIT-TO (PUBYEAR , 2012) OR LIMIT-TO (PUBYEAR , 2011) OR LIMIT-TO (PUBYEAR , 2010) OR LIMIT-TO (PUBYEAR , 2009) OR LIMIT-TO (PUBYEAR , 2008) OR LIMIT-TO (PUBYEAR , 2007) OR LIMIT-TO

(PUBYEAR , 2006) OR LIMIT-TO (PUBYEAR , 2005) OR LIMIT-TO (PUBYEAR , 2004) OR LIMIT-TO (PUBYEAR , 2003) OR LIMIT-TO (PUBYEAR , 2002) OR LIMIT-TO (PUBYEAR , 2001) OR LIMIT-TO (PUBYEAR , 2000) OR LIMIT-TO (PUBYEAR , 1999) OR LIMIT-TO (PUBYEAR , 1998) OR LIMIT-TO (PUBYEAR , 1997) OR LIMIT-TO (PUBYEAR , 1996) OR LIMIT-TO (PUBYEAR , 1995) OR LIMIT-TO (PUBYEAR , 1994) OR LIMIT-TO (PUBYEAR , 1993) OR LIMIT-TO (PUBYEAR , 1992) OR LIMIT-TO (PUBYEAR , 1991) OR LIMIT-TO (PUBYEAR , 1990))

PsycINFO.

1 ((psychother* or counsel*) and (supervis* or trainee* or training)).ti.
2 (psychother* or therap*).ti,ab.
3 (psychotherapist* or psychologist* or psychiatrist* or therapist* or professional* or clinician* or counsellor* or counselor* or personnel* or employee* or practioner* or supervisor*).ti.
4 (supervis* or counseling or counselling or tutor* or mentor* or guiding or guidance or trainee* or placement* or training or praxis* or teach* or skill* or knowledge or competen* or performanc* or learn* or educat* or qualificat* or (professional adj development) or (therapist adj development) or (therapist's adj development) or career* or (personal adj practice) or (clinical adj practice) or ((development or developing) and practice*) or (practice adj setting) or (practice adj settings) or experienc*).ti.
5 2 and 3 and 4
6 1 or 5
7 limit 6 to yr="1990 -Current"
8 limit 7 to english language
9 limit 8 to "0400 dissertation abstract"
10 8 not 9

Cochrane Central Register of Controlled Trials. Searced via “EBM Reviews - Cochrane Clinical Answers”, part of Ovid's Evidence Based Medicine Reviews collection.

1 (psychother* OR therapist* OR councelor* OR councellor* OR supervisor* OR psychologist* OR psychiatrist*),ti
2 (supervis* OR trainee* OR training OR counseling or counselling).ti
3 1 and 2
4 limit 3 to yr="1990 -Current"
5 limit 4 to english language
6 5 use cctr

Restricting result with shortname – “cctr” (set 6), to identify articles in Cochrane Central Register of Controlled Trials
