# Supplementary material for: Learning Processes and Acquisition of Knowledge and Skills in Training and Supervision of Psychotherapy and Counselling: A Study Protocol for a Scoping Review
Source: Front Psychol. 2021 Dec 16;12:718314. doi: 10.3389/fpsyg.2021.718314 (PMC8716550; doi:10.3389/fpsyg.2021.718314)
Supplement: Supplementary file 2 [file Table_1.pdf]

Appendix B

Eligibility screening form
Scoping review on psychotherapy supervision and training and learning processes in

		**yes**	**no**
**1.**	Involves psychotherapy training or supervision		
**2.**	If yes, is it empirical (and specify whether quantitative, qualitative, or mixed-method)		
**3.**	If yes, is it conceptual		
**4.**	If yes, is it program training description		
5.	If yes, is it a review, meta-analysis/synthesis,		
